# Predictive outcome of late window ischemic stroke patients following endovascular therapy: a multi-center study

**DOI:** 10.3389/fneur.2025.1489714

**Published:** 2025-03-27

**Authors:** Sijie Zhou, Mohammad Mofatteh, Zhikai Chen, Jinyan Tang, Jicai Ma, Ziqi Ouyang, Jian Wang, Senrong Luo, Yuankang He, Zhenzhang Li, Yuzheng Lai, Xuxing Liao

**Affiliations:** ^1^Department of Surgery of Cerebrovascular Diseases, First People’s Hospital of Foshan, Foshan, China; ^2^School of Medicine, Dentistry and Biomedical Sciences, Queen’s University Belfast, Belfast, United Kingdom; ^3^Graduate School, Guangdong Medical University, Zhanjiang, China; ^4^Department of Neurosurgery, First People’s Hospital of Foshan, Foshan, China; ^5^The First Clinical Medical College, Guangdong Medical University, Guangdong Medical University, Zhanjiang, China; ^6^Department of Neurology, The Affiliated Yuebei People’s Hospital of Shantou University Medical College, Shaoguan, China; ^7^Department of Neurosurgery, Advanced National Stroke Center, Foshan Sanshui District People’s Hospital, Foshan, China; ^8^College of Mathematics and Systems Science, Guangdong Polytechnic Normal University, Guangzhou, China; ^9^School of Basic Medical Sciences, Guangzhou Medical University, Guangzhou, China; ^10^Department of Neurology, Guangdong Provincial Hospital of Integrated Traditional Chinese and Western Medicine (Nanhai District Hospital of Traditional Chinese Medicine of Foshan City), Foshan, China

**Keywords:** ischemic stroke, large vessel occlusion, endovascular therapy, thrombectomy, patient outcome, late time window used machine learning

## Abstract

**Background and purpose:**

Stroke is a leading cause of morbidity and mortality worldwide. Endovascular therapy (EVT) has been established as a gold standard option to treat acute ischemic stroke (AIS) patients with large vessel occlusion (LVO) presenting within 6 h of symptom onset. However, there is a paucity of information regarding patient outcome and mortality in patients presenting in late time window within 6 to 24 h. In this study, we aimed to assess for predictors of outcomes in late window stroke patients following EVT.

**Methods:**

We analyzed data from 202 patients treated with EVT from four comprehensive stroke centers. All patients were above 18 years of age and had symptoms onset of 6–24 h. mRS of 0–2 after three months was defined as favorable outcome.

**Results:**

Patients with favorable outcome had lower median age (*p =* 0.003), lower pre-EVT National Institute of Health Stroke Scale (NIHSS) score (*p =* 0.000), lower diabetes mellitus (*p =* 0.041), stroke history (*p =* 0.041), parenchymal hematoma (PH) (*p =* 0.000) and fewer attempts to achieve successful recanalization (*p =* 0.001). Multivariate regression analysis found age (*p =* 0.007), diabetes mellitus (*p =* 0.022), successful recanalization (mTICI≥2b) (*p =* 0.006), NIHSS at onset (*p =* 0.000), and PH1 + PH2 Heidelberg bleeding classification (*p =* 0.009) as predictors of functional outcome.

**Conclusion:**

Age, diabetes mellitus history, baseline NIHSS score, successful recanalization, and PH are predictors of 90-day functional outcome of late-window ischemic stroke patients undergoing EVT.

## 1 Introduction

Stroke is a major cause of disability worldwide with estimated 12.2 million annual incident cases and the second leading cause of death resulting in 6.55 million annual mortalities (1). Several landmark randomized clinical trial publications revealed endovascular therapy (EVT) as a safe and effective treatment which can be offered as a gold-standard for suitable acute ischemic stroke (AIS) patients with large vessel occlusion (LVO) presenting within 6 h of symptoms onset (2, 3). Significant randomized clinical trials, DEFUSE-3 and DAWN, published in 2018 which demonstrated that EVT is also highly effective in patients presented in late time window between 6 to 24 h after symptomatic onset (4, 5). Therefore, it is believed that EVT can be extended as a gold-standard treatment approach for patients presenting between 6 and 24 h as well. Recently, predicting the outcome of patients in stroke medicine has gained momentum to establish predictive models to facilitate the provision of personalized medicine (6, 7).

In this study, we aimed to predict the outcome and mortality of ischemic stroke patients presenting in late time window (6–24 h) who underwent EVT.

## 2 Methods

### 2.1 Patient population

In this retrospective study, we enrolled prospectively collected data of consecutive AIS patients with LVO who underwent EVT at four comprehensive stroke centers in China. We derived our data from the Big Data Observatory Platform for stroke in China in addition to individual hospital data platforms.

Inclusion criteria for this study were as follows as established previously (8): (1) modified Rankin Scale (mRS) score before onset: 0–2; (2) interval from onset or the final onset-free time: 6–24 h; (3) age ≥ 18 years old, pre-EVT (National Institute of Health Stroke Scale) NIHSS score ≥ 6; (4) involved with internal carotid artery (ICA), tandem (ICA + M1), M1, M2, or basilar artery occlusion. Patients with missing follow-up and last know well to puncture time ≤ 6 h were excluded.

### 2.2 Data collection

We collected the following data in our study: age, sex, risk factors of vascular diseases, such as hypertension, diabetes mellitus, coronary artery disease (CAD), atrial fibrillation (AF), dyslipidemia, previous stroke history, initial premorbid mRS, pre-EVT NIHSS, time metrics, site of arterial occlusion, modified thrombolysis in cerebral infarction (mTICI) post thrombectomy. Successful revascularization was defined as TICI 2b to 3 (8) and complete reperfusion was defined as mTICI of 3.

The patients’ outcomes were evaluated by using mRS 3-month after EVT was performed. Favorable outcome was defined as mRS of 0–2 after three months, as explained previously (9, 10).

### 2.3 Statistical analysis

The non-parametric Mann–Whitney U test was performed using IBM SPSS 26 version (IBM-Armonk, NY) to analyze non-normally distributed continuous data, reported as medians along with the interquartile range (IQR) in our study. Normally distributed data were reported as means with corresponding standard deviations (SD) and compared using the student’s t-test. Results were considered statistically significant if the *p*-value was less than 0.05.

### 2.4 Ethics statement

The Institutional Review Board of all hospitals involved in the study approved the study protocol. All procedures in the study involving human participants were performed in accordance with the ethical standards of the institutional and/or national research committee and with the 1964 Declaration of Helsinki and its later amendments or comparable ethical standards. Written informed consents from the participants’ legal guardian/next of kin were obtained to perform the EVT.

## 3 Results

**Figure 1:**
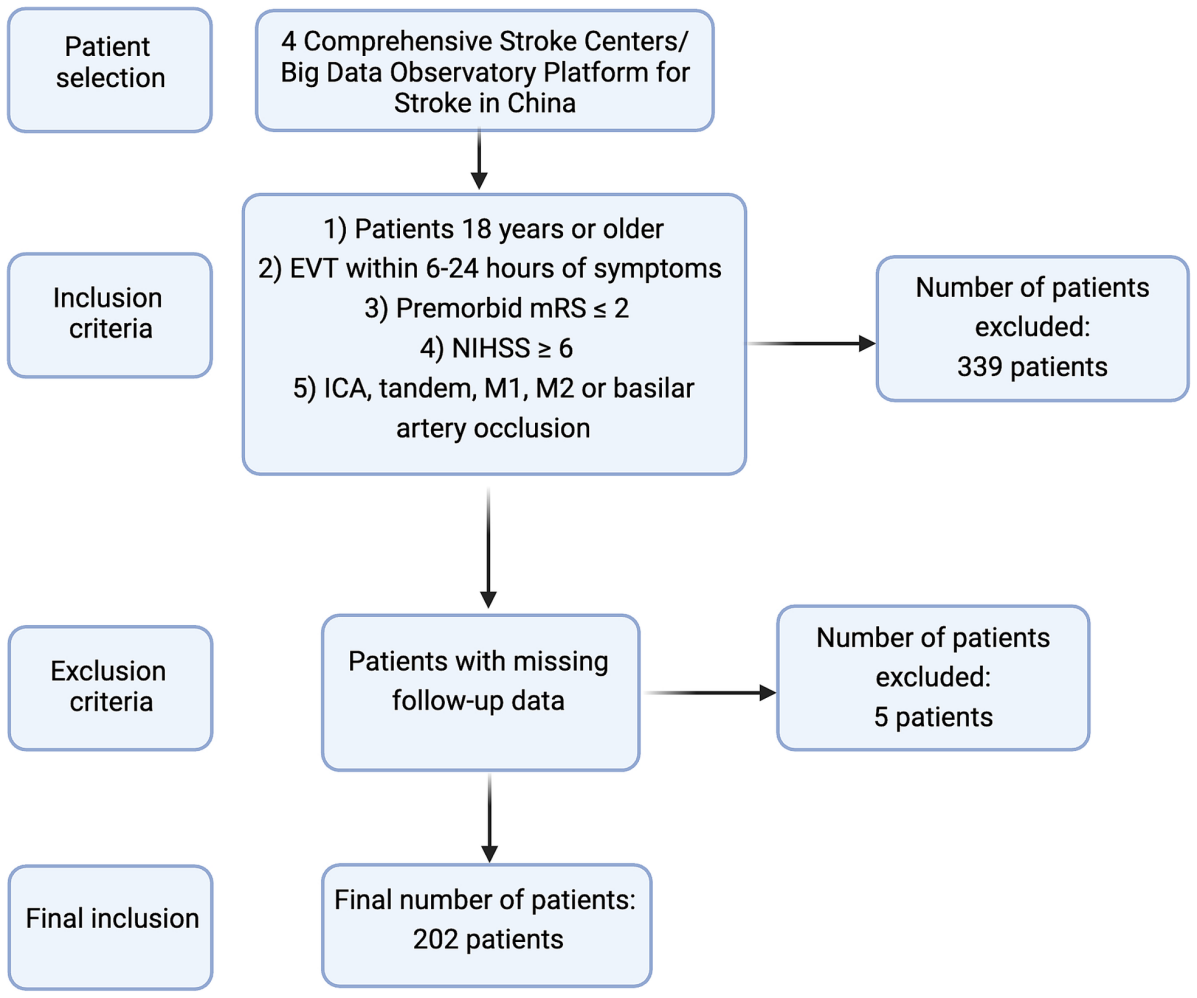
A summary of patient selection criteria in the current study.

We treated a total of 541 consecutive AIS patients. After applying the inclusion and exclusion criteria, a final cohort of 202 patients were included in the final analysis. In total, 322 patients were excluded due to last known normal-to-puncture-time (LKNPT) of within 6 h. Seven patients had occlusions which did not involve ICA, Tandem (ICA + M1), M1, M2, and basilar artery. Five patients were missed to follow-up and five other patients had pre-EVT NIHSS <6. A summary of patients’ selection criteria is demonstrated in Figure 1.

The baseline characteristics of patients are demonstrated in Table 1. Patients with favorable outcome had 7 years lower median age (*p =* 0.003) and had a median 4 points lower pre-EVT NIHSS (*p =* 0.000). In addition, the favorable outcome group had lower diabetes mellitus (*p =* 0.041), stroke history (*p =* 0.041), and PH1 + PH2 Heidelberg bleeding classification (*p =* 0.000). Furthermore, fewer attempts were required to achieve successful recanalization in the favorable outcome patient group (*p =* 0.001).

**Table 1:** Baseline characteristics of patients in favorable and unfavorable outcome groups.

	Unfavorable outcome	Favorable outcome	*X*^2^/z	*P*
Number	115	87		
Age (median, IQR)	68.0 (56.0, 75.0)	61.0 (50.0, 70.0)	−3.001	0.003**
Female sex, *n*, %	35 (30.43)	24 (27.59)	0.194	0.659
Hypertension, *n*, %	82 (71.30)	56 (64.37)	1.101	0.294
Diabetes mellitus, *n*, %	31 (26.96)	13 (14.94)	4.196	0.041*
Coronary artery diseases, *n*, %	21 (18.26)	10 (11.49)	1.746	0.186
Atrial fibrillation, *n*, %	36 (31.30)	17 (19.54)	3.542	0.060
Previous stroke, *n*, %	31 (26.96)	13 (14.94)	4.196	0.041*
Dyslipidemia	28 (24.35)	25 (28.74)	0.493	0.483
Pre-EVT NIHSS (median, IQR)	18.0 (14.0, 22.0)	14.0 (10.0, 18.0)	−4.398	0.000**
ASPECTS pre-treatment (median, IQR)	8.0 (7.0, 8.0)	8.0 (7.0, 9.0)	−1.778	0.075
ICA	19 (16.52)	13 (14.94)	6.078	0.299
M1	46 (40.00)	42 (48.28)		
M2	5 (4.35)	6 (6.90)		
ICA + M1	19 (16.52)	17 (19.54)		
Basilar	22 (19.13)	8 (9.20)		
Others	4 (3.48)	1 (1.15)		
Intravenous thrombolysis, *n*, %	18 (15.65)	12 (13.79)	0.135	0.713
LKNPT (median, IQR), min	580.0 (450.0, 825.0)	615.0 (446.0, 861.0)	−0.605	0.545
Successful recanalization (mTICI ≥ 2b)	90 (78.26)	83 (95.40)	11.836	0.001**
Parenchymal hematoma	24 (20.87)	3 (3.45)	12.982	0.000**

ASPECTS, Alberta stroke program early CT score; EVT, endovascular treatment; IQR, interquartile range; LKNPT, last known normal-to-puncture-time; mTICI, modified thrombolysis in cerebral infarction; NIHSS, National Institute of Health Stroke Scale Score.*Denotes significance.

There were no statistically significant differences in sex (*p =* 0.659), hypertension (*p =* 0.294), coronary artery disease (*p =* 0.186), dyslipidemia (*p =* 0.483), pre-treatment Alberta stroke programme early CT score (ASPECTS) (*p =* 0.075), occlusion site (*p =* 0.299), intravenous thrombolysis (*p =* 0.713), LKNPT (*p =* 0.545) between the unfavorable and favorable outcome groups.

Our multivariate regression analysis showed that age (*p =* 0.007), diabetes mellitus (*p =* 0.022), successful recanalization (mTICI≥2b) (*p =* 0.006), NIHSS at onset (*p =* 0.000), and PH1 + PH2 Heidelberg bleeding classification (*p =* 0.009) (Table 2). In contrast, other factors such as atrial fibrillation (*p =* 0.500), previous stroke history (*p =* 0.216), initial pre-treatment ASPECTS (*p =* 0.120) were not associated with favorable outcome.

**Table 2:** Multivariate Regression Factors Associated with Favorable Outcome.

Variables	Coefficient	SE	*z*	Wald χ^2^	*p*	OR	OR of 95% CI
Age	−0.044	0.016	−2.698	7.281	0.007*	0.957	0.927 ~ 0.988
Diabetes mellitus	−0.953	0.415	−2.298	5.279	0.022*	0.385	0.171 ~ 0.869
Atrial fibrillation	0.290	0.429	0.675	0.455	0.500	1.336	0.576 ~ 3.097
Previous stroke	−0.528	0.427	−1.238	1.533	0.216	0.590	0.256 ~ 1.361
successful recanalization (mTICI ≥ 2b)	1.729	0.624	2.770	7.675	0.006*	5.633	1.658 ~ 19.139
Pre-EVT NIHSS	−0.113	0.029	−3.907	15.267	0.000*	0.893	0.844 ~ 0.945
Pre-EVT ASPECTS	0.250	0.160	1.556	2.422	0.120	1.283	0.937 ~ 1.757
Parenchymal hematoma	−1.765	0.675	−2.614	6.833	0.009*	0.171	0.046 ~ 0.643

ASPECTS, Alberta stroke program early CT score; mTICI, modified thrombolysis in cerebral infarction; NIHSS, National Institute of Health Stroke Scale Score. *Denotes significance.

## 4 Discussion

Despite significant advances in stroke care over the past decades, the prognosis of acute ischemic stroke patients has been a major challenge for clinicians worldwide, therefore establishing methods to predict functional outcome of patients undergoing EVT is of great clinical importance. In the present study, we aimed to predict the outcome and mortality of ischemic stroke patients from four comprehensive stroke centers presenting in late time window (6–24 h) who underwent EVT. Our data revealed that multiple factors, including age, diabetes mellitus history, successful recanalization (mTICI≥2b), NIHSS at onset, and PH1 + PH2 Heidelberg bleeding classification were associated with favorable outcome in patients with late-presentation who underwent EVT. However, atrial fibrillation, previous stroke history, and initial pre-treatment ASPECTS were not associated with favorable outcome.

Conducting multivariate regression analysis showed that only age, diabetes mellitus, successful recanalization (mTICI≥2b), NIHSS at onset, and PH1 + PH2 Heidelberg bleeding classification were factors involved in the prediction of favorable patient outcome. In comparison, our multivariate regression analysis did not show atrial fibrillation, previous stroke history, initial pre-treatment ASPECTS as factors predictive of favorable outcome. It is worth emphasizing that while ASPECTS was not associated with favorable outcome, initial pre-treatment ASPECTS was shown to be an important factor in our multivariate regression analysis.

A large multi-center prospective study of 1708 patients in France reported that patient age was strongly associated with 90-day functional outcome in patients treated with EVT with LKNPT fewer than 8 h, which is consistent with our findings (11). Our data is aligned with other published studies in European centers, disseminating such information from Asian centers can expand and validate the previously published studies.

Diabetes mellitus diagnosis was another factor in our multivariate regression analysis which was crucial for predicting favorable patient outcome. Similar to our results, a large multi-center European study of 695 patients revealed that diabetes mellitus diagnosis was an independent factor to predict poor functional outcome and mortality after 90 days in stroke patients (12). In addition to human investigations, multiple studies on animal have also reported that a larger infarct area was associated with elevated serum glucose level compared to animals with normal blood glucose (13, 14). Furthermore, some studies proposed multiple mechanisms to explain that more severe tissue damage was observed in AIS patients with diabetes mellitus, including intracellular acidosis, accumulation of extracellular glutamate, disruption to the blood–brain barrier, development of cerebral edema, and impairment of plasma fibrinolysis (15, 16).

Consistent with findings in our investigation, another prospective multi-center study also reported baseline NIHSS correlations with unfavorable functional outcomes in AIS patients treated EVT (17). NIHSS is a critical and well-established tool for determining the risk of clinical deterioration to assess the stroke severity to guide treatment decision-making, thereby selecting suitable treatment options, particularly before decision to initiate EVT (17).

Successful recanalization was another predictor of functional outcome revealed in our study. A recent large-scale European study comprised of 5,853 patients with AIS presenting in late time-window also reported that successful vessel recanalization was independently associated with a higher rate of favorable outcomes, defined as a mRS score of 3 or lower in their investigation (18).

Novel methods in future investigations are required to address the existing challenges in stroke treatment. Accurate stroke diagnosis and determining the exact time of onset are crucial components of comprehensive stroke management. However, a major challenge for clinicians remains predicting the outcomes for stroke patients, including radiological outcomes (e.g., hemorrhagic transformations, final infarct volume), morbidity (e.g., stroke-associated pneumonia), mortality, and other functional independence indices (e.g., mRS, cognitive functions, language functions, Barthel Index score) (19). Machine learning, a subfield of artificial intelligence, utilizes computerized algorithms that automatically improve performance through iterative learning or experience gained from data acquisition (19). As technological advances continue to transform medicine, machine learning has proven especially useful in establishing diagnoses, planning treatments, and predicting outcomes in patients with AIS (20–22). These approaches can analyze vast amounts of clinical and laboratory data within short time frames and with high accuracy, providing valuable insights into stroke research and treatment (23). Machine learning has been employed to identify suitable candidates for specific treatments or to predict patients likely to experience poor functional outcomes at 90 days (24, 25). Various machine learning approaches, including support vector machine (SVM), linear discriminant analysis (LDA), k-nearest neighbors (KNN), and decision tree algorithms have recently been employed in clinical research, paving the way for using large-scale data to improve clinical decision-making and patient prognosis (26–30). Therefore, establishment and utilization of a machine learning algorithm in our study can prompt other neurology and neurosurgery centers to adopt similar approaches for EVT.

A major advantage of our study is its multi-center nature across four large comprehensive stroke centers from an under-studied population in China. Our study has some limitations. First, this was a retrospective study with a medium sample size. We emphasize that prospective multi-center studies are required to validate these findings. Second, some factors, including final infarct volume, and arterial re-occlusion rates were not included in our analysis. In addition, predictive factors obtained in the study were not cross-validated, which is an important consideration for future investigations. Furthermore, we were unable to analyze data on Intracranial atherosclerotic disease (ICAD), intracranial stent or angioplasty use, as well as extracranial stent use. It is also worth emphasizing that we used the ASPECTS to analyze mainly, due to the limited data on computed tomographic perfusion (CPT) and magnetic resonance perfusion (MRP) to calculate the exact core volume in our study. A sensitivity analysis should be conducted in future studies by including the study center as a covariate in multivariate model. Our analysis showed no significant inter-center differences after adjusting for key clinical factors. Nevertheless, our results can prompt other stroke centers to investigate the outcome of patients presenting in late-window undergoing EVT.

## 5 Conclusion

Age, diabetes mellitus history, baseline NIHSS score, successful recanalization, and PH1 + PH2 Heidelberg bleeding classification are prognostic tools for the prediction of favorable outcomes in AIS patients presenting between 6 and 24 h of symptoms onset undergoing EVT. Future studies in other patient population and geographical areas are required to validate and extend these findings.

## Data Availability

The raw data supporting the conclusions of this article will be made available by the authors, without undue reservation.
